# Developing GM insects for sustainable pest control in agriculture and human health

**DOI:** 10.1186/1753-6561-8-S4-O43

**Published:** 2014-10-01

**Authors:** Glen Slade, Neil Morrison

**Affiliations:** 1 Oxitec Ltd, 71 Milton Park, Oxford OX14 4RQ, UK

## 

With increasing demand for effective control of insect pests coupled with environmental sustainability, farmers and disease vector control authorities are facing enormous challenges to, respectively, maintain food production and protect human health. Pest control has long relied upon insecticides, which can be very effective, but continued reliance is hampered by several factors. Concerns over their potential impacts on the environment and human health have led to implementation of restrictions on residues on food, the number of sprays per season, implementation of spray-free pre-harvest periods, and withdrawal from the market of some modes of action. Insect populations develop resistance to insecticides, and there are a limited range of modes of action available. These issues have driven the development of diverse other pest control tools - such as mating disruption using sex pheromones, release of natural predators, spraying biopesticides (e.g. *Bt*) and cultivation of insect-resistant transgenic crops - which can be employed together to form integrated pest management (IPM) strategies. We propose a new pest control approach, called RIDL (Release of Insects carrying a Dominant Lethal) [1], as a potentially valuable new IPM component for agriculture and public health.

RIDL utilises transgenic technology to engineer novel traits in pest insects, for application against the wild pest population. We have generated RIDL strains in several insect species: in the dengue vector mosquito *Aedes aegypti *[2], for example, when larvae are reared in restrictive conditions, male and female offspring do not survive to adulthood due to over-expression of a lethal effector gene, tTA. Permissible conditions are provided by adding tetracycline to the larval medium: the 'Tet-off' genetic system, and therefore expression of tTA, is suppressed and the insects survive as normal. Releasing these insects into the wild over a sustained period leads to mating between released males and wild females, resulting in population suppression as their progeny do not survive in the absence of tetracycline. In the factory, however, the RIDL strain can be reared as normal with tetracycline. These transgenic insect strains also express a fluorescent protein marker, which is heritable, easily screened under specialised filters and robust in field conditions.

This approach is similar in effect to another mating-based pest control strategy, the Sterile Insect Technique (SIT), in which mass-reared insects are sterilised by radiation prior to release into the field. SIT (and RIDL) offers pest control that is highly species-specific, with consequently minimal ecological impact; and as it relies upon the mate-seeking instincts of male insects, it is highly effective against low-density or difficult-to-reach pest populations, and can provide highly effective local pest eradication and a barrier to reinvasion. SIT has been used with success against a number of important pest insects, notably in eradicating the New World Screwworm (*Cochliomyia hominivorax*) from North and Central America. Despite its success, wider SIT implementation is constrained by several inherent limitations. The use of radiation to sterilise the insects also compromises their performance in the field, and the requirement to invest in costly radiation sources and facilities generally restricts SIT application to large-scale programmes that justify the investment. Release of both sexes of sterile insects reduces SIT efficiency - with Mediterranean fruit fly (Medfly, *Ceratitis capitata*), male-only releases are 3-5 × more efficient per male than are bi-sex releases [3]- and generating sexing strains that permit efficient, large-scale sex-sorting is technically challenging by conventional chromosomal translocation methods. Rearing and releasing large numbers of a pest insect requires a reliable method of marking the methods, to distinguish between wild and sterile, and presents the risk of accidental escapes of fertile, mass-reared pests.

RIDL overcomes these challenges, providing an alternative to sterilisation by irradiation, built-in bio-containment, and a heritable visible genetic marker. Moreover, a female-specific variant of RIDL, called fsRIDL, offers a means of producing male-only cohorts of the insects on a large scale. These have been developed in the Tephritid fruit flies olive fly (*Bactrocera oleae*) and Medfly, and the Lepidoptera diamondback moth (*Plutella xylostella*) and pink bollworm (*Pectinophora gossypiella*), using sex-alternate splicing sequences from sex determination genes (*transformer *and *doublesex *in the Tephritids and Lepidoptera, respectively) to regulate female-specific expression of the tTA effector gene, conferring tetracycline-repressible lethality in females only [4-6]. After fsRIDL males are released into the field they find and mate with wild females, the female progeny of which do not survive: as with RIDL and the SIT, with sustained releases the reproductive capacity of the wild population crashes.

Several of these RIDL/fsRIDL strains have undergone further assessment for potential application in the field. Laboratory experiments have been conducted to characterise traits relevant to future field performance, such as longevity, male mating competitiveness and penetrance of the engineered trait when reared on natural host plants (compared to artificial diets). Protocols for these experiments have typically been developed and validated for the SIT, particularly with Tephritid fruit flies. On-crop survival and mating competitiveness trials have been conducted in field cages. Later experiments, conducted in large cages in greenhouses, have sought to investigate whether releases of fsRIDL insects will suppress a target population, as designed. In these cages, wild-type populations of the target pest - olive flies [6], Medfly or diamondback moth(manuscripts in preparation) - were established and stabilised, after which weekly releases of fsRIDL males were initiated and the population size monitored relative to those in untreated cages. In all instances, caged populations treated with fsRIDL males crashed to extinction.

In addition to directly suppressing a wild pest population, fsRIDL also provides a powerful insecticide resistance management benefit [7]. Survival of fsRIDL males results in the background genetics of the mass-reared insect population introgressing into the target population. If the mass-reared fsRIDL colony comprises insecticide susceptibility alleles, the resulting introgression into the wild pest population leads to a powerful reduction in the population's resistance to a given chemical mode of action. There is, therefore, scope for synergistic use of fsRIDL and insecticides, with potential to reduce overall insecticide use and protect efficacy of valuable and effective modes of action.

In the open field, the first trials with a transgenic strain of insect was with a fluorescent protein-marked strain of pink bollworm, irradiated and used for conventional SIT in Arizona, USA [8]. This trial provided evidence that genetically engineered insects can perform well in the field compared to wild-type insects, and assessment of the strain for programmatic SIT use is ongoing.

In a small town in the Cayman Islands, releases of male RIDL *Ae. aegypti *mosquitoes (strain 'OX513A') were conducted in 2009 and 2010 [9,10]. Relative trap captures of RIDL and wild mosquitoes, together with larvae of each genotype hatching from eggs collected from the field in ovitraps, indicated that they could perform strongly in terms of finding and competing for wild mates in the field. Male-only releases, principally to avoid additional biting by released female mosquitoes (male mosquitoes do not blood-feed), were facilitated by a pupal sex-sorting method that separates males and females by size (females are bigger). In 2010, releases of RIDL males were conducted over a more prolonged period, resulting in suppression of the wild population by 80% relative to nearby untreated areas. Following this first demonstration of RIDL efficacy in the field, OX513A has undergone open field trials in Malaysia and Brazil, where further success has been demonstrated.

This genetic technology shows great promise for species-specific and powerful control of the dengue vector mosquito, *Ae. aegypti*, and other difficult-to-control and important pests such as Medfly, olive fly, pink bollworm, diamondback moth and *Ae. albopictus*. Furthermore, cross-species function of RIDL and fsRIDL systems - in Tephritid fruit flies, mosquitoes and Lepidoptera - demonstrates that this technology should be relatively easily transferred to other target species in the future, offering a new pest control tool for wider implementation of IPM in agriculture and public health.

References
